# Patterns in the Microbial Community of Salt-Tolerant Plants and the Functional Genes Associated with Salt Stress Alleviation

**DOI:** 10.1128/Spectrum.00767-21

**Published:** 2021-10-27

**Authors:** Yanfen Zheng, Zongchang Xu, Haodong Liu, Yan Liu, Yanan Zhou, Chen Meng, Siqi Ma, Zhihong Xie, Yiqiang Li, Cheng-Sheng Zhang

**Affiliations:** a Marine Agriculture Research Center, Tobacco Research Institute of Chinese Academy of Agricultural Sciences, Qingdao, China; b Department of Ocean Science and Engineering, Southern University of Science and Technology, Shenzhen, China; c National Engineering Laboratory for Efficient Utilization of Soil and Fertilizer Resources, College of Resources and Environment of Shandong Agricultural University, Taian, China; d School of Life Sciences, Ludong University, Yantai, China; USDA—San Joaquin Valley Agricultural Sciences Center

**Keywords:** functional genes, functional signatures, metagenomics, microbiome, saline soil, salt-tolerant plant

## Abstract

Salinity is an important abiotic stress affecting plant growth. We have known that plants can recruit beneficial microbes from the surrounding soil. However, the ecological functions of the core microbiome in salt-tolerant plants, together with their driving factors, remain largely unexplored. Here, we employed both amplicon and shotgun metagenomic sequencing to investigate the microbiome and function signatures of bulk soil and rhizocompartment samples from three salt-tolerant plants (legumes Glycine soja and Sesbania cannabina and nonlegume Sorghum bicolor). Strong filtration effects for microbes and functional genes were found in the rhizocompartments following a spatial gradient. The dominant bacteria belonged to *Ensifer* for legumes and *Bacillus* for *S. bicolor*. Although different salt-tolerant plants harbored distinct bacterial communities, they all enriched genes involved in cell motility, Na^+^ transport, and plant growth-promoting function (e.g., nitrogen fixation and phosphate solubilization) in rhizoplane soils, implying that the microbiome assembly of salt-tolerant plants might depend on the ecological functions of microbes rather than microbial taxa. Moreover, three metagenome-assembled genomes affiliated to *Ensifer* were obtained, and their genetic basis for salt stress alleviation were predicted. Soil pH, electrical conductivity, and total nitrogen were the most important driving factors for explaining the above microbial and functional gene selection. Correspondingly, the growth of an endophyte, Ensifer meliloti CL09, was enhanced by providing root exudates, suggesting that root exudates might be one of factors in the selection of rhizosphere and endosphere microbiota. Overall, this study reveals the ecological functions of the populations inhabiting the root of salt-tolerant plants.

**IMPORTANCE** Salinity is an important but little-studied abiotic stressor affecting plant growth. Although several previous reports have examined salt-tolerant plant microbial communities, we still lack a comprehensive understanding about the functional characteristics and genomic information of this population. The results of this study revealed the root-enriched and -depleted bacterial groups, and found three salt-tolerant plants harbored different bacterial populations. The prediction of three metagenome-assembled genomes confirmed the critical role of root dominant species in helping plants tolerate salt stress. Further analysis indicated that plants enriched microbiome from soil according to their ecological functions but not microbial taxa. This highlights the importance of microbial function in enhancing plant adaptability to saline soil and implies that we should pay more attention to microbial function and not only to taxonomic information. Ultimately, these results provide insight for future agriculture using the various functions of microorganisms on the saline soil.

## INTRODUCTION

Soil salinization is an immense problem for agricultural production worldwide. Planting salt-tolerant plants is found to effectively improve soil properties. Microbiota inhabiting the different spatial niches of these plants have important roles in enhancing host resistance to salt stress (1, 2) and promoting productivity (3). In turn, plants provide a variable but large amount of carbon through photosynthesis for these surrounding microorganisms (4). Thus, salt-tolerant plants and their associated microbiota formed a complex and dynamic interaction during long-term coevolution.

Plant-growth-promoting rhizobacteria (PGPR) have excellent root colonizing and enzyme production abilities. It is well established that salt-tolerant plants have special root microbiota containing a wide range of PGPRs (5, 6). Indeed, some PGPRs are demonstrated to have the ability to alleviate the salt effect on plant growth and promote plant adaptation to salinity (7–10). For example, Yasmin et al. found that Pseudomonas pseudoalcaligenes and Bacillus subtilis significantly improved the growth of soybean under salt stress through a series of physiological regulatory processes (11). At present, the known mechanisms (10, 12) involved in microorganisms promoting plant growth under saline soil may include the following: producing 1-aminocyclopropane-1-carboxylic acid (ACC) deaminase and indole-3-acetic acid (IAA) to improve plant growth for indirectly reducing salt stress for plants, synthesizing phosphatase and siderophore to provide available nutrients for plants, activating the antioxidative systems of plants to scavenge reactive oxygen species (ROS), and producing exopolysaccharide to facilitate biofilm formation and mediate ionic balance through binding Na^+^. However, the above mechanisms are illustrated only in several microbial species, and their distributions in the whole microbial community of salt-tolerant plants are unknown.

Microbiome assemblage can be affected by many factors, including plant genotypes, developmental stages, soil types, land management practices, and geographical locations (13). An in-depth understanding of plant microbiome members and functions under saline soil is an essential step toward their application in agriculture. Many previous studies have demonstrated that a large number of microbes associated with salt stress adaptation were enriched in the rhizosphere of salt-tolerant plants (6, 14, 15). These rhizobiomes have been extensively studied to reveal their important roles in plant adaptation to salinity (11, 16, 17). For example, Yuan et al. found that Pseudomonas species, the core rhizospheric bacteria of the halophyte plant, can improve the salt tolerance of nonhalophyte plants (14). Xiong et al. revealed that Bacillus flexus KLBMP 4941, recruited by the coastal halophyte Limonium sinense, can promote host plant growth under salt stress (18). However, most previous studies of salt-tolerant plant-associated microbial communities were conducted by means of ribosomal amplicon-based approaches, which only provide taxonomic information of the microbiome. Recently, shotgun metagenomic sequencing that can provide taxonomic, genomic, and functional information for a given community has been widely applied to the soil and rhizosphere microbiome studies of barley (19), citrus (20), wheat (21), cucumber (21), and Jerusalem artichoke (5). By comparison, to date, we still know little about the genomic and functional contents of salt-tolerant plant microbiota.

Leguminous plants are one of the most diverse lineages that can develop root nodules and fix nitrogen by rhizobia (22). They are reported to secrete different root exudates from nonlegumes in ingredient and quantity (23). These root exudates are generally used as signal molecules and nutrients for soil microbes (24). The legumes, Glycine soja and Sesbania cannabina, which naturally live in the saline soils of the Yellow River Delta (China), can tolerate high salt. Our previous study found a higher number of rhizobia in the root nodule of these two legume plants compared with that of the cultivated legume, Glycine max (25). However, the microbiome assemblages and microbial functions of the two salt-tolerant legumes are not yet clear. The plant microbiome at later growth stages has been revealed to harbor higher microbial diversity and more stress-tolerant bacteria (26). Thus, in this study, we collected bulk soil, rhizosphere soil, rhizoplane soil, and root from two legumes (*G. soja* and *S. cannabina*) and one nonlegume (Sorghum bicolor) at the plant mature stage, and performed amplicon and deep shotgun metagenomic sequencing. We aimed to (i) determine the dominant microbial populations across the salt-tolerant plant species, (ii) evaluate the ecological functions of microbial communities in the salt-tolerant plants, and (iii) explore the driving factors that influence the root-associated microbial community and functional gene assemblies.

## RESULTS

### Variation of microbial α-diversity and microbial community composition among the three salt-tolerant plants.

**FIG 1:**
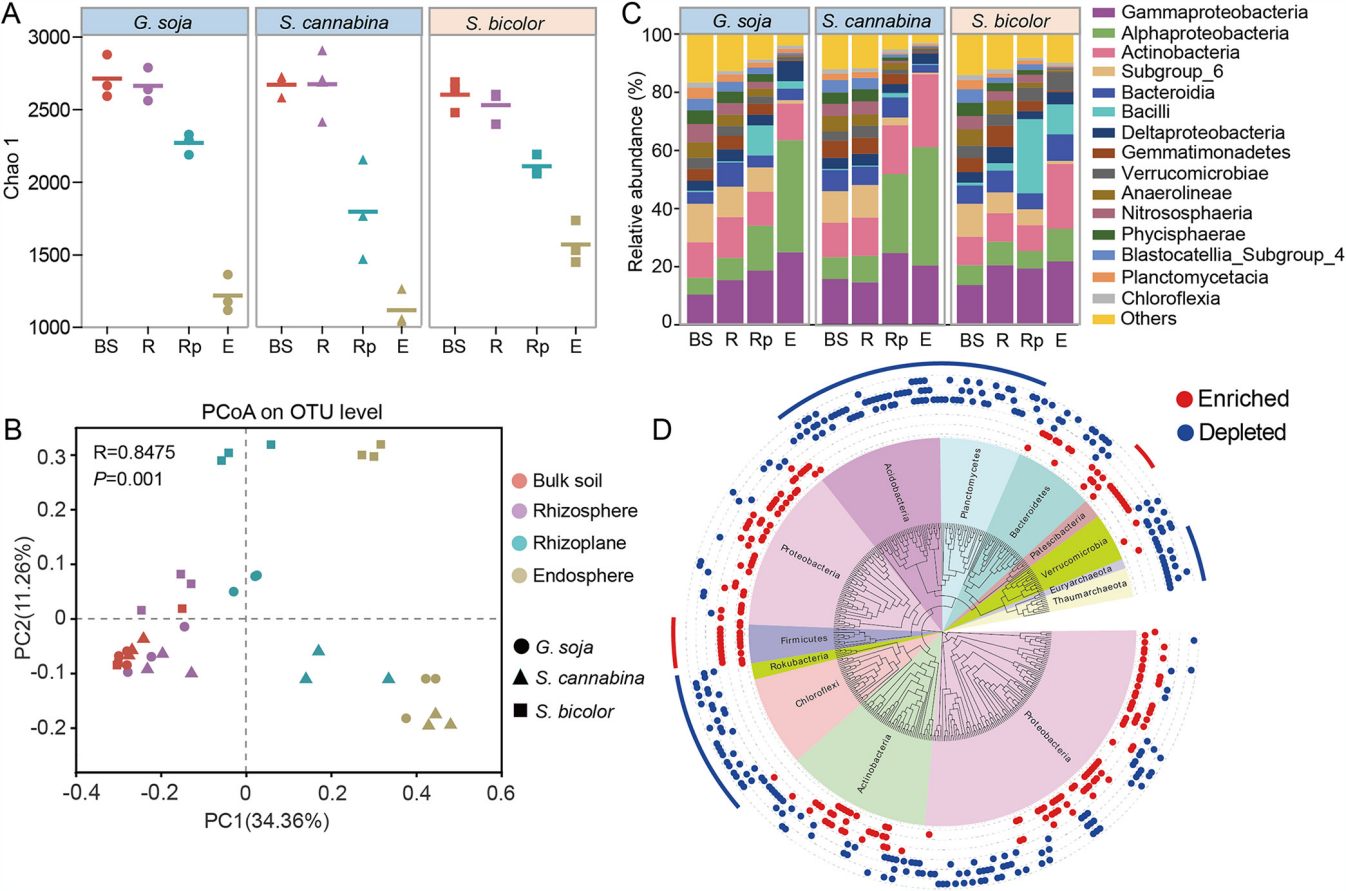
Bacterial diversity and community of the three salt-tolerant plants. (A) A decreasing gradient in microbial diversity revealed by Chao 1 from the bulk soil to the endosphere. (B) PCoA of all samples based on the Bray-Curtis distance. (C) The bacterial relative abundance of each plant compartment at the phylum level. (D) Phylogenetic tree of endosphere or rhizoplane-enriched and -depleted OTUs. The inner colored ring represents the phylum that each OTU belongs to. The middle ring of the colored dots represents the enriched (red) or depleted (blue) OTUs compared with those in bulk soil. The same-colored dots from the inner to outer rings represent *G. soja*, *S. cannabina*, and *S. bicolor*. The lines in the outer ring indicate that nearly all OTUs in the phylum are either enriched (red) or depleted (blue). BS, bulk soil; R, rhizosphere; Rp, rhizoplane; E, endosphere.

Legume (*G. soja* and *S. cannabina*) and nonlegume (*S. bicolor*) plants were planted in coastal saline soil (see Fig. S1 in the supplemental material). We found an α-diversity gradient from bulk soil to the endosphere both in legume and nonlegume plants (Fig. 1A; see also Fig. S2 in the supplemental material). The bulk soil had the highest α-diversity, which was similar to that of the rhizosphere soil (*P* > 0.05). In contrast, the root endosphere had the lowest α-diversity among all compartments (*P* < 0.05; see Table S1 in the supplemental material).

Principal coordinates analysis (PCoA) revealed that the rhizocompartments were separated clearly by the first principal coordinate (PC1, 34.36%), and plant species were separated by the second principal coordinate (PC2, 11.26%) (Fig. 1B). Permutational multivariate analysis of variance (PERMANOVA) confirmed that compartment niche (*R^2^* = 52.31%; *P < *0.001) had a larger contribution to the microbiome variation than plant species (*R^2^* = 12.03%; *P < *0.05) based on the Bray-Curtis distance metric (see Table S2 in the supplemental material). A volcano plot showed that the enriched operational taxonomic unit (OTU) number increased gradually from rhizosphere to endosphere in all three plants (see Fig. S3 in the supplemental material), demonstrating a strong filtration effect from the outer to inner layers of the plant root. Compared to bulk soil, all *Firmicutes* and *Patescibacteria* OTUs were enriched in endosphere and/or rhizoplane, whereas nearly all OTUs in *Thaumarchaeota* (archaea), *Acidobacteria*, *Planctomycetes*, *Chloroflexi*, and “*Candidatus* Rokubacteria” were depleted (Fig. 1D; see also Fig. S4 in the supplemental material).

The bacterial abundance at the class level indicated notable differences across the plant species (Fig. 1C). *Alphaproteobacteria*, *Gammaproteobacteria*, *Actinobacteria*, and *Acidobacteria* subgroup 6 were dominant groups in all three plants regardless of compartments, accounting for 39.8 to 86.7% of the whole community. However, different plants had distinct bacterial taxonomic profiles. Specifically, the endosphere microbiome of two legumes, *G. soja* and *S. cannabina*, had a greater proportion (*P* < 0.05) of *Alphaproteobacteria* (38.51 to 40.82%) than *S. bicolor* (11.23%). While *S. bicolor* harbored large amounts of *Bacilli* with a proportion of 10.38%, which was higher (*P* < 0.01) than that in *G. soja* and *S. cannabina* (0.31 to 2.55%) (Fig. 1C; see also Table S3 in the supplemental material).

### Dominant bacteria among different compartments and plants.

**FIG 2:**
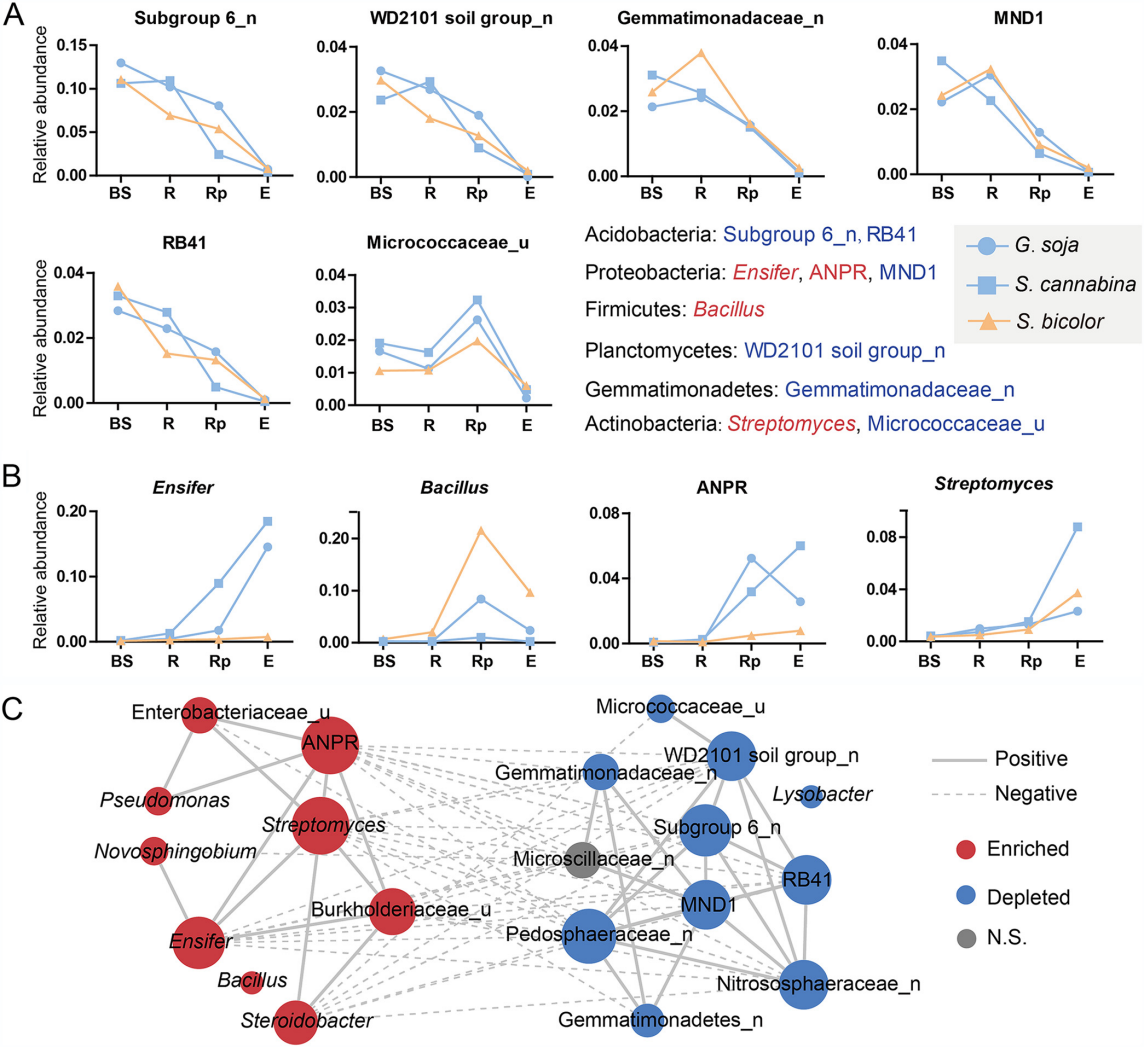
Distribution of the top 20 abundant genera in different compartments and salt-tolerant plants. Significantly depleted (A) and enriched (B) microbes in the endosphere compared with other compartments. (C) Network visualization of the interaction among the top 20 abundant genera. Blue words in panels A and B indicate that the microbes have previously only been described by environmental sequences or their members are difficult to cultivate. The suffixes “n” and “u” indicates “no rank” and “unclassified,” respectively. BS, bulk soil; R, rhizosphere; Rp, rhizoplane; E, endosphere; ANPR, *Allorhizobium*-*Neorhizobium*-*Pararhizobium*-*Rhizobium*.

The microbial communities were further analyzed at the genus level, and all top 10 abundant genera were found to be either enriched or depleted in the endosphere. Six of the 10 were significantly depleted, i.e., subgroup 6, WD2101 soil group (*Planctomycetes*), family of *Gemmatimonadaceae*, MND1 (*Proteobacteria*), RB41 (*Acidobacteria*), and unclassified family *Micrococcaceae* (Fig. 2A). Another four microbes, namely, *Ensifer*, *Bacillus*, *Allorhizobium*-*Neorhizobium-Pararhizobium-Rhizobium* (ANPR), and *Streptomyces* were significantly enriched (Fig. 2B). The linear discriminant analysis (LDA) effect size (LEfSe) confirmed that subgroup 6 was the enriched bacteria for bulk soil and *Ensifer* for root (see Fig. S5A in the supplemental material). A network was constructed to investigate the possible interactions between the enriched and depleted genera. Intriguingly, two obvious clusters were obtained. The enriched genera in the rhizoplane and/or endosphere microbiome showed negative associations with the depleted genera, whereas both intracluster associations were positive (Fig. 2C).

Different salt-tolerant plants were found to possess distinct dominant bacteria. Rhizobia (*Ensifer* and ANPR) were exclusively increased in the rhizoplane and endosphere of legume plants, whereas *Bacillus* was more abundant in *S. bicolor* (Fig. 2B). Additionally, *Novosphingobium* (5.1%) and the order of “*Candidatus* Saccharimonadales” (3.6%) with quite higher proportion were observed in the endosphere of legumes and *S. bicolor*, respectively (see Fig. S6 in the supplemental material). Correspondingly, the LEfSe further determined that *Ensifer* and *Novosphingobium* were enriched in legumes and *Bacillus* and “*Candidatus* Saccharimonadales” in *S. bicolor* (see Fig. S5B).

### Relationships between microbial communities and soil properties.

**FIG 3:**
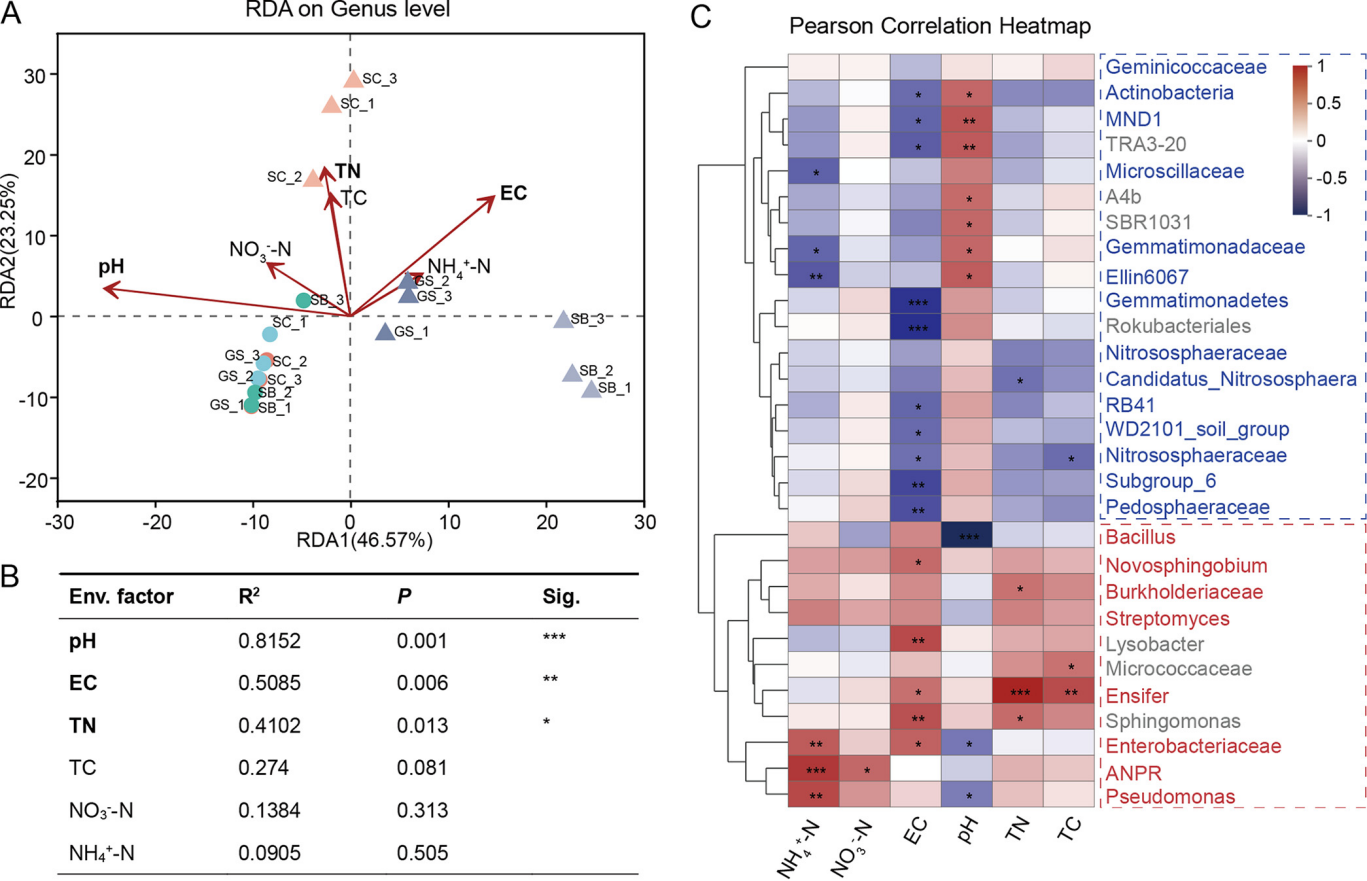
Correlations between microbial abundance and soil physicochemical factors. (A) RDA plots showing the relationships between soil physicochemical factors and genus-level community structures and the related statistical tests shown in panel B. Circles and triangles in panel A represent bulk soil and rhizoplane soil, respectively. (C) Pearson correlation analyses were performed between the top 30 abundant genera and the physicochemical factors. The red and blue colors indicate positive and negative correlations, respectively. Different significance levels of correlation analyses are marked with asterisks (*, *P* < 0.05; **, *P* < 0.01; ***, *P *< 0.001).

The redundancy analysis (RDA) model (Fig. 3A) and Monte Carlo permutation test (Fig. 3B) were employed to examine the relationship between the five soil physicochemical properties and bacterial community structure. According to the RDA results, pH was the most important factor influencing bacterial community structure (*R*^2^ = 0.815; *P *= 0.001), followed by electrical conductivity (EC) (*R*^2^ = 0.509; *P *= 0.006) and total nitrogen (TN) (*R*^2^ = 0.410; *P = *0.013). pH showed a negative correlation with rhizoplane soil samples, whereas EC and TN showed a contrasting tendency (Fig. 3A). The top 30 abundant genera were separated into two clusters based on soil physicochemical properties (Fig. 3C). One cluster contained rhizoplane soil-enriched genera, which positively correlated with EC and TN, while another cluster containing rhizoplane soil-depleted genera, which showed a negative correlation with pH.

### Taxonomic and functional category in rhizocompartments revealed by metagenomics.

**FIG 4:**
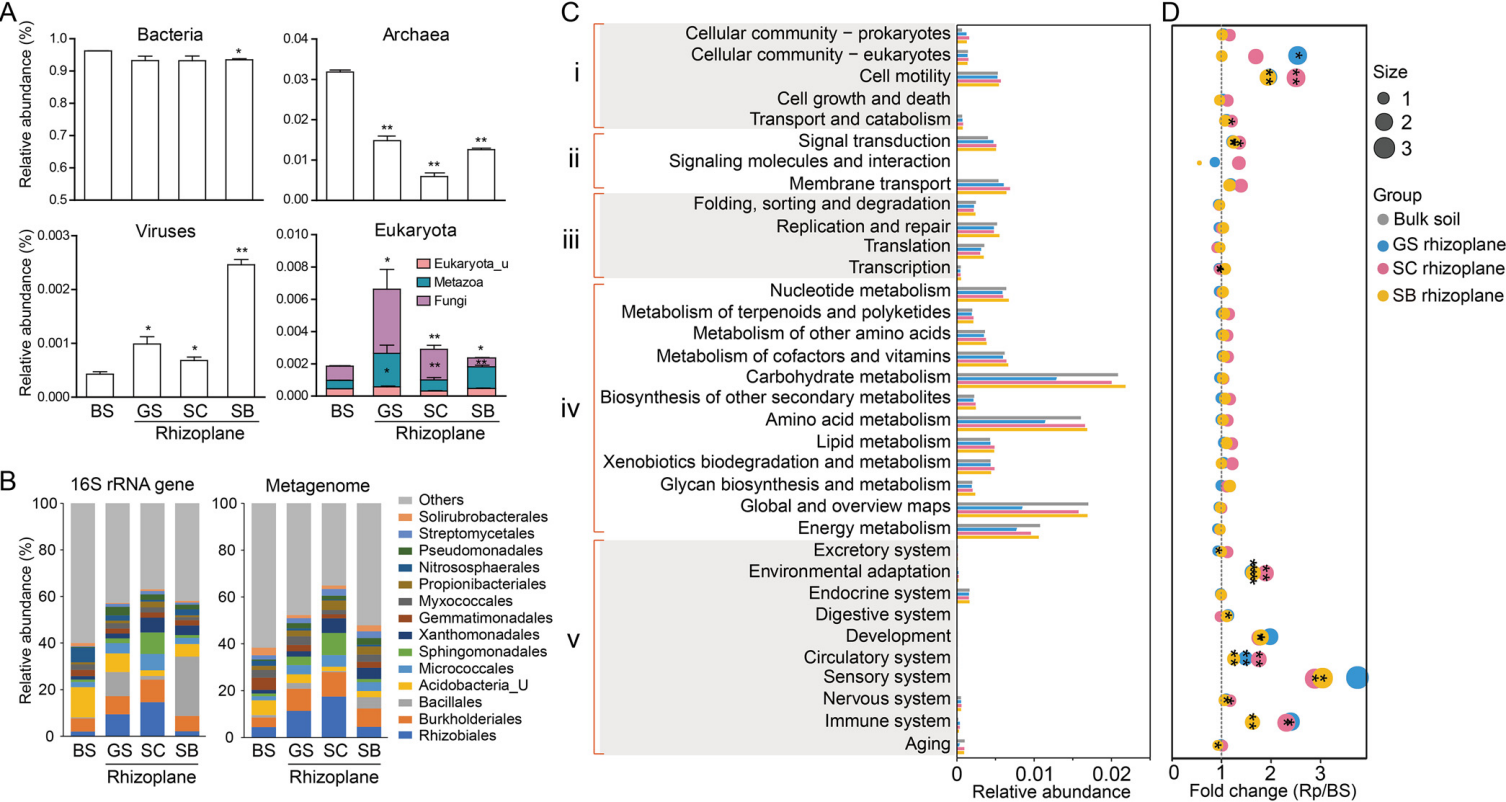
Taxonomic and functional gene profiles revealed by metagenomics. (A) The distribution of bacteria, archaea, viruses, and eukaryota in bulk soil and rhizoplane soil. Significant difference was tested between bulk soil and each rhizoplane soil. (B) Comparison of order-level distributions based on 16S rRNA gene amplicon and metagenomic data. (C) Relative abundances of genes affiliated to KEGG pathways in bulk soil and rhizoplane soils. Roman numerals i to v indicate cellular processes, environmental information processing, genetic information processing, metabolism, and organismal systems, respectively. (D) Fold change in relative abundance between bulk soil and rhizoplane soil. Pathways significantly enriched in bulk soil or rhizoplane soil are marked with asterisks. *, *P *< 0.05; **, *P* < 0.01; ***, *P *< 0.001. BS, bulk soil; GS, *G. soja*; SC, *S. cannabina*; SB, *S. bicolor*.

To determine the functions of enriched microbial populations in the rhizoplane, independent Illumina shotgun sequencing was performed. As no significant difference was found among the bacterial communities of the three bulk soils using analysis of similarities (ANOSIM) (*P* = 0.307), only the bulk soil sample from *G. soja* was selected as the comparison object for cost-effective purposes. Taxonomic analysis of the metagenomic data based on the NCBI-nr database revealed that bacteria accounted for the majority of the soil populations, with 93.1 to 93.5% in rhizoplane and 96.2% in bulk soil. Archaea, viruses, and eukaryotes only accounted for a small proportion and inhabited different niches. Specifically, viruses and eukaryotes (mainly fungi and metazoa) were enriched in rhizoplane soil, while archaea were depleted, compared with that in the bulk soil (Fig. 4A). Comparison of 16S rRNA gene amplicon and metagenome sequencing revealed a similar taxonomic profile, although several bacterial lineages, such as *Bacillales*, were overrepresented in the 16S rRNA gene taxonomic profiling (Fig. 4B). Such differences may be attributed to the fact that *Bacillales*, belonging to the phylum of *Firmicutes*, were reported to possess multiple 16S rRNA gene copies, with an average copy number of 6.0 (27).

According to the metagenomic data annotated against the KEGG database, the relative abundance of categories of cell motility, signal transduction, and environmental adaptation were significantly higher (*P *< 0.05) in the rhizoplane soil than bulk soil (Fig. 4C). Cell motility is the important functional trait involved in known plant-microbe and microbe-microbe interactions, which might serve as critical factors for the assemblage of rhizosphere microbiomes and plant colonization (13). Thus, genes assigned to cell motility were further analyzed, and we found that nearly all genes in this category were higher in rhizoplane soil than bulk soil (see Fig. S7 in the supplemental material). Methyl-accepting chemotaxis protein (KEGG entry K03406) was the most abundant protein related to cell motility in the rhizoplane. Only one among the top 30 proteins, i.e., the two-component system, chemotaxis family, CheB/CheR fusion protein (KEGG entry K13924), was enriched in the bulk soil. The major source organism for cell motility was the phylum of *Acidobacteria* in the bulk soil. However, different source organisms were observed among the three rhizoplane soils. The cell motility genes in legume plants were mainly derived from *Ensifer* and *Novosphingobium*, while *Bacillus* was the major source organism for *S. bicolor* (see Fig. S8 in the supplemental material).

### The functional genes that potentially alleviate salt stress for plants.

**FIG 5:**
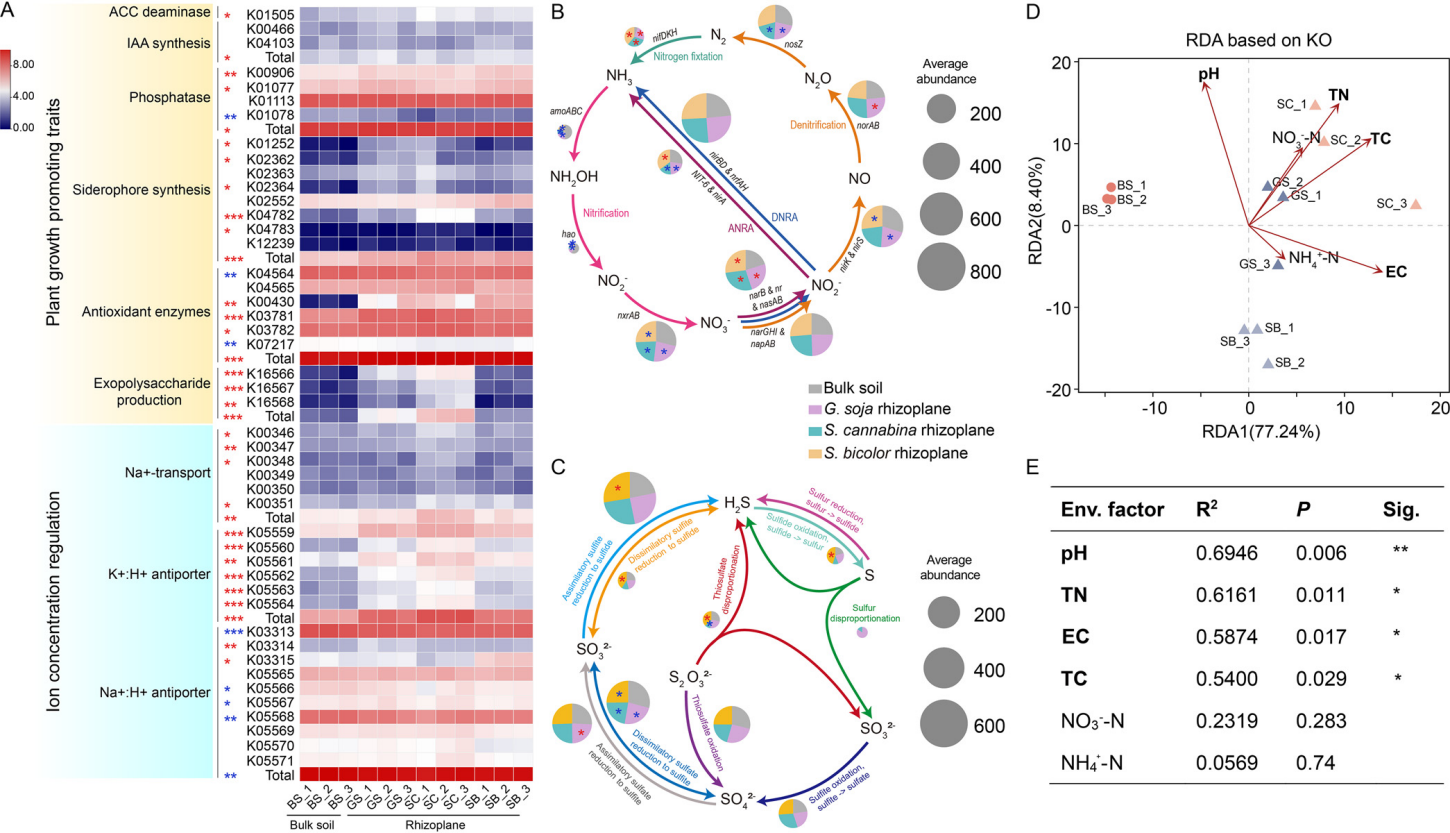
The distribution of genes involved in plant growth promotion (A), nitrogen (B), and sulfur (C) cycles and the correlations between the KEGG ortholog (KO) group and soil physicochemical factors (D, E). Red and blue stars indicate that gene abundance is higher or lower in the rhizoplane soils than bulk soil, respectively. The star number in panel A represents plant number with significant difference between rhizoplane and bulk soil for this gene. The size of the pie chart in panels B and C is proportional to the relative abundance of the gene involved in the pathway. DNRA, dissimilatory nitrate reduction to ammonia; ANRA, assimilatory nitrate reduction to ammonia. Circles and triangles in panel D represent bulk soil and rhizoplane soil, respectively. Different significance levels of correlation analyses (E) are marked with asterisks (*, *P *< 0.05; **, *P* < 0.01). BS, bulk soil; GS, *G. soja*; SC, *S. cannabina*; SB, *S. bicolor*.

To explore the potential function of rhizoplane microbiota in alleviating the effect of salt on plant growth, we searched for genes related to plant-growth-promoting (PGP) traits and ion concentration regulation. The results showed that compared to bulk soil, microbiota in rhizoplanes (at least one plant) possessed a higher proportion of genes involved in PGP traits, including ACC deaminase, IAA biosynthesis, phosphatase, siderophore synthesis, antioxidant enzymes, and exopolysaccharide production (Fig. 5A). The relative abundances of genes involved in Na^+^-transport and K^+^/H^+^ antiporter, reported to directly reduce salt toxin in the plant, were higher in the rhizoplanes than bulk soil, whereas Na^+^/H^+^ antiporter genes showed an opposite trend. Microbes can also promote plant growth through nutrient production. Genes responsible for the nitrogen fixation pathway were more abundant in the rhizoplane than bulk soil, while most processes related to the nitrification and denitrification pathways were more abundant in bulk soil (Fig. 5B), leading to the accumulation of nitrogen in rhizoplane soil. This finding was consistent with the higher TN concentration observed in rhizoplane soil (see Table S4 in the supplemental material). For sulfur cycling, only the relative abundance of genes related to the process of dissimilatory sulfate reduction to sulfite was lower in the three rhizoplanes, and sporadic difference was observed in other processes (Fig. 5C).

We reconstructed 39, 45, 40, and 32 metagenome-assembled genomes (MAGs) from bulk soil, *G. soja* rhizoplane, *S. cannabina* rhizoplane, and *S. bicolor* rhizoplane soils, respectively. However, we did not find any nitrogen fixation pathway in all bulk soil MAGs, while three or four MAGs were found to contain this pathway in each of the three rhizoplane soils (see Table S5 in the supplemental material). Two MAGs encoding ACC deaminase and four MAGs encoding phosphatase were found in bulk soil, which were lower than that found in rhizoplane soil, with 2 to 7 and 6 to 11 MAGs, respectively. The RDA model (Fig. 5D) and Monte Carlo permutation test (Fig. 5E) revealed that pH, TN, EC, and total carbon (TC) were the main factors driving the functional gene variation between the bulk soil and rhizoplane soil of salt-tolerant plants.

### Genomic information of three MAGs from legume plants.

**FIG 6:**
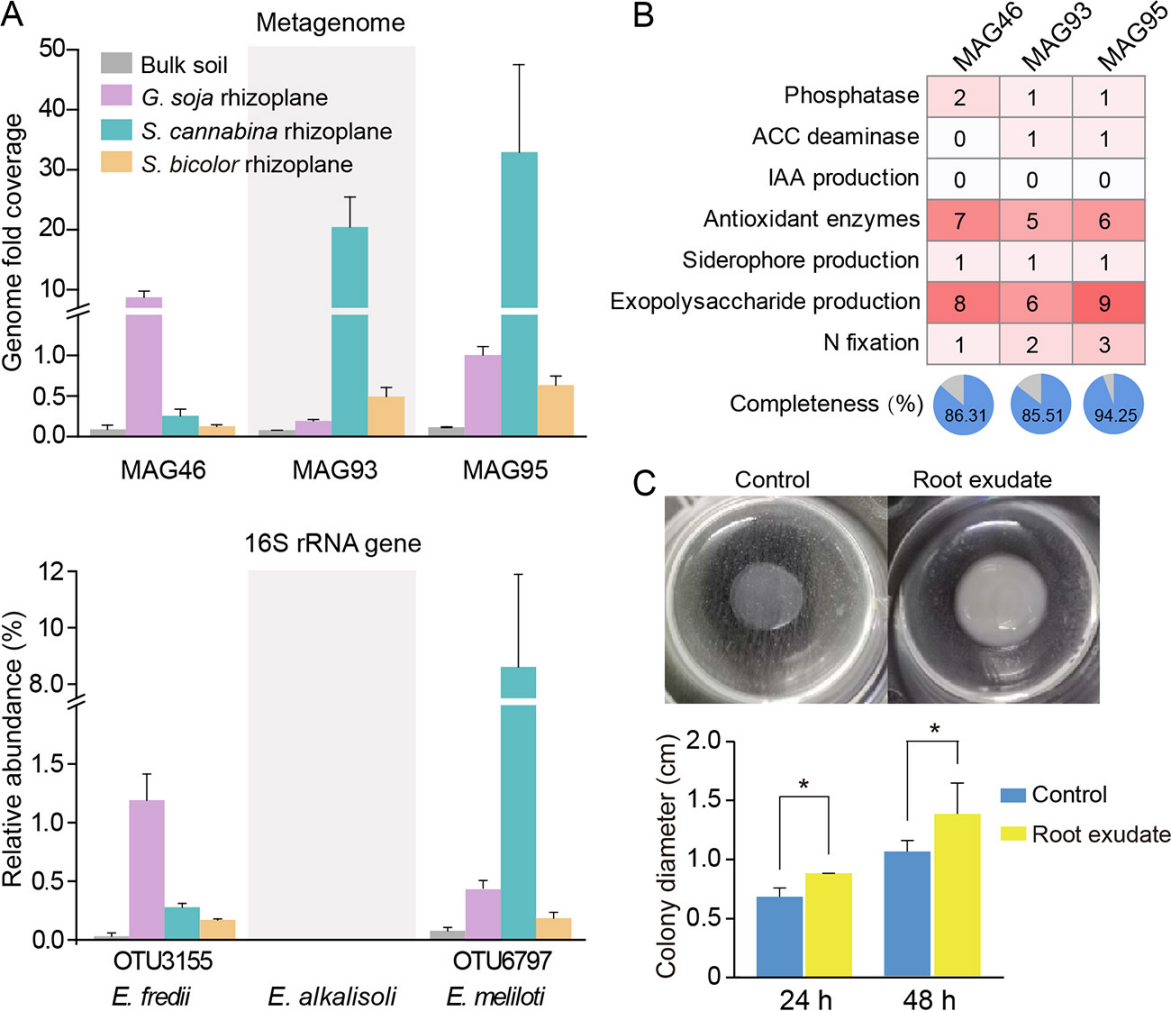
The relative abundance and potential functional features of the core microbes of legume plants. (A) Relative abundance of core microbes in bulk soil and rhizoplane soils revealed by metagenome and 16S rRNA gene amplicon sequencing. (B) Gene numbers in MAG46, MAG93, and MAG95 related to potential plant growth promotion. (C) Colony diameter of *E. meliloti* CL09 with or without root exudate. This experiment was conducted with three biological replicates.

To better understand the putative functions of dominant bacteria in legume plants, we further analyzed the genomic information of three MAGs affiliated to *Ensifer*, termed MAG46 (completeness of 86.31%), MAG93 (completeness of 85.51%), and MAG95 (completeness of 94.25%). These MAGs were identified as Ensifer fredii, Ensifer alkalisoli, and Ensifer meliloti using Genome Taxonomy Database (GTDB), with G+C contents of 63.3%, 62.5%, and 62.3%, respectively. All reads were mapped to their genomes, and the results showed that they displayed higher genome fold coverage in legume plants. There were 34- and 69-fold as many reads matching *E. fredii* MAG46 in the *G. soja* rhizoplane as those in the *S. cannabina* and *S. bicolor* rhizoplanes, respectively (Fig. 6A), suggesting that MAG46 was the predominant bacteria in *G. soja*. The number of reads matching the *E. alkalisoli* MAG93 and *E. meliloti* MAG95 genomes was up to 106- and 52-fold higher in *S. cannabina* than *G. soja* and *S. bicolor* rhizoplanes, respectively, indicating that these two MAGs were core microbes in *S. cannabina*. Consistently, the 16S rRNA gene amplicon analysis also revealed a predominance of *E. fredii* (OTU3155) and *E. meliloti* (OTU6797) in *G. soja* and *S. cannabina*, respectively, although none of the OTUs related to *E. alkalisoli* were found (Fig. 6A).

To determine the potential roles played by the above three *Ensifer* MAGs on *G. soja* or *S. cannabina* rhizoplanes, all genes were classified into 25 subsystem functional categories. Most of the genes were associated with functions, such as metabolisms of amino acids and derivatives, carbohydrates, and respiration (see Table S6 in the supplemental material). Further, these three *Ensifer* MAGs were found to possess a series of genes associated with cell motility and plant growth promotion ability, including phosphatase, ACC deaminase, antioxidant enzymes, production of exopolysaccharide and siderophore, and nitrogen fixation (Fig. 6B).

To explore how legumes enriched *Ensifer* species from bulk soil, we isolated 50 axenic rhizobial strains on yeast mannitol agar (YMA) medium. Most isolates affiliated to *Ensifer*, including *E. meliloti* CL09, were isolated from *S. cannabina*. This isolate showed 100% 16S rRNA gene similarity to the most abundant OTU sequences (OTU6797) derived from high-throughput amplicon sequencing, indicating that it represents an indigenous species in *S. cannabina*. We collected the root exudate of *S. cannabina* and found that this isolate could grow better when root exudate was provided (Fig. 6C).

## DISCUSSION

An increasing number of studies have investigated the plant microbiota composition (19, 28–30). However, the microbial communities of salt-tolerant plants and functional genes associated with salt stress alleviation are still unclear. The data presented here provide a deeper characterization of the salt-tolerant plant microbiome, involving the microbial community structure and functional gene profile. Our results showed that the endosphere microbiomes of salt-tolerant plants are shaped by host selection. Metagenomic analysis revealed that genes involved in cell motility, PGP traits (e.g., phosphate solubilization), and Na^+^ concentration regulation were enriched in rhizoplane soil compared with that in bulk soil, and this variation was driven by soil pH, EC, and TN. To the best of our knowledge, this is the first study to evaluate the functional gene distributions of salt-tolerant plants using metagenomic analyses, greatly enhancing our understanding in microbial assembly mechanisms and their ecological importance in plant adaptability to saline soil.

In our study, bacterial diversity decreased from the rhizosphere across the rhizoplane to the root endosphere, which is likely the result of multiple filtering processes by plant (31). Although *Acidobacteria* subdivisions (32) and representatives from *Thaumarchaeota* (33) have been reported to have the potential to promote plant growth and/or protect against abiotic stress, numerous OTUs belonging to *Acidobacteria* and *Thaumarchaeota* depleted gradually from the rhizosphere to endosphere in this study. As it is difficult or even impossible to cultivate the members of these two phyla, their potential function has mainly been described through environmental sample sequencing. Further studies are required to determine why plants in this study did not enrich microbes in these two phyla. Above differences in microbial community structure between bulk soil and rhizoplane soil might be explained by pH, EC, and TN, which reflected that microbes inhabiting different niches had distinct ecological adaption strategies.

*Ensifer* was previously found in a legume plant under saline soil (25, 34). Genomic and ecological studies have focused on *Ensifer* species due to their unique roles in nodule formation, nitrogen fixation, and agricultural use for soil remediation (28, 34, 35). In this study, two legume plants *G. soja* and *S. cannabina* harbored different *Ensifer* OTUs, with the reported broaden-host-range species *E. fredii* (OTU3155) (36) and narrow-host-range species *E. meliloti* (OTU6797) (37) dominant in the root endosphere, respectively. Different from legume plants, *Bacillus* was the dominant bacteria in *S. bicolor*. Previous studies have revealed that some species of *Ensifer* (38) and *Bacillus* (11, 18) could improve plant salt tolerance, implying the central role of dominant bacterial populations. In order to further determine their function in resisting salinity stress, microbial communities and functions of the salt-tolerant plants in unstressed soil should be considered as a control in future study. Additionally, we found that most taxa depleted in the endosphere were these microbes: they have only been described based on environmental sequences or their members are difficult to cultivate. However, the enriched microbes are general heterotrophic bacteria that can be cultivated by common methods, which is perhaps due to the fact that the resources supplied in culturing medium are more similar to those in the rhizosphere environment than in bulk soil.

Most reports, as in this study, have revealed that genes involved in cell motility (e.g., chemotaxis and flagella assembly) were highly abundant in the rhizosphere and/or endosphere microbial communities (20, 39, 40) as well as bacterial isolate genomes (41). Chemotaxis is used by plant-associated microorganisms to sense signals (e.g., organic acids) from root exudates. Once a signal is perceived, microorganisms move toward the plant through the flagella and form a biofilm to initiate colonization (13). In this study, cell motility genes were mainly derived from the rhizoplane core microbes, i.e., *Ensifer* and *Novosphingobium* in legume plants and *Bacillus* in *S. bicolor*. Thus, we envision that the strong motility allows these microbes from bulk soil to rapidly arrive at their preferred root surface niches and form active microbe-plant interactions. This speculation was supported by the fact that the root exudate of legume plant stimulated the growth of *Ensifer* species in this study. It should be noted that root exudate may only provide nutrients for bacterial growth. Their chemoattractant effect on core bacteria and specific chemical composition should be further comprehensively analyzed.

Many plant-associated microbes, especially PGPR, have ACC deaminase, IAA biosynthesis, phosphatase, siderophore production, nitrogen fixation, and antioxidant enzymes, which can improve the salt tolerance of their host (10). In this study, genes encoding proteins involved in the above processes were more abundant in the rhizoplane than bulk soil, suggesting that plants preferred to enrich microbes having the potential to help them alleviate salt stress. The Na^+^/H^+^ antiporter is the known system for Na^+^ extrusion (42). The lower abundance of the Na^+^/H^+^ antiporter gene in rhizoplane microbes would make them retain Na^+^ in the cell. As the salt tolerance of the microorganism is markedly higher than that of plant cells, the accumulated Na^+^ would not influence their growth. Thus, root-associated microbes might protect their host from high Na^+^ concentrations this way. Such a conjecture was in agreement with previous studies that mycorrhizal fungi decreased Na^+^ concentration in shoot tissue of plants through accumulating Na^+^ in their intraradical hyphae (43, 44). Correspondingly, we found that the abundance of the K^+^/H^+^ antiporter gene was higher in the rhizoplane soil than bulk soil. The K^+^/H^+^ antiporter is responsible for expelling K^+^ from the cell (45). The higher abundance of this gene in rhizoplane microbes would increase the content of K^+^ available for plant uptake, resulting in a high ratio of K^+^/Na^+^. This high K^+^/Na^+^ ratio could prevent the disruption of various enzymatic processes and the inhibition of protein synthesis in plants under salt stress (46). This might be another strategy for root microbes to help their host alleviate salt stress. Future experimental evidence is required to elucidate this mechanism, such as where Na^+^ is retained in the rhizoplane microbes and how the microbes regulate their K^+^/Na^+^ ratio.

In this study, we revealed the diversity, assembly, and functional gene profile of the root-associated microbial communities of three salt-tolerant plants. We found that bulk soil and rhizoplane compartments exhibit different taxonomic and functional gene patterns. Microbiota in the salt-tolerant plant rhizoplane soils possessed more abundant genes associated with plant growth promotion and salt stress alleviation than bulk soil. Correspondingly, the reconstructed genome from metagenomic reads revealed the genetic potential in alleviating salt stress for plants. We further found that the microbial community and functional gene filtration by salt-tolerant plants were influenced by soil pH, EC, and TN. It is important that future studies of the salt-tolerant plant microbiome consider more sampling time points since the microbiome assembly is influenced by general stoichiometry and dynamic environmental conditions, including the plant growth stages, soil salinity, and climate.

## MATERIALS AND METHODS

### Experimental field.

Experiments were performed at the modern agriculture technology experiment and demonstration base of Shandong Academy of Agricultural Sciences, located at Dongying city (37°18′7.62″N, 118°37′13.24″E), Shandong, China. Soil of the experimental area in this study has an EC of ∼600 μs/cm and pH 8.7. The seeds of three examined plants (*G. soja*, *S. cannabina*, and *S. bicolor*) were sown at the end of April 2019. Each plant was planted in three individual areas (∼100 m^2^ for each) without fertilization.

### Sample collection.

Samples were collected on 30 September 2019 when the plants were in the mature growth stage (see Fig. S1 in the supplemental material). For each plant, samples collected from three points were combined to generate one composite sample. Sterile gloves were worn when samples were collected. Soil distanced from root ∼20 cm was sampled and considered as bulk soil (see Fig. S9 in the supplemental material). The rhizosphere and rhizoplane soil samples were collected as reported by Edwards et al. (47). Then, roots were soaked in alcohol (75%) for 2 min and sodium hypochlorite (5%) for 5 min. Final rinse was performed three times with sterile water and 1 min for each. We referred to the community in surface-sterilized root as the endosphere microbiome. All samples were stored at −80°C until DNA extraction. In total, 36 samples, including three plants (*G. soja*, *S. cannabina*, and *S. bicolor*), with four compartments (bulk soil, rhizosphere, rhizoplane, and endosphere) and three replicates for each were collected.

### Measurement of the soil properties.

The soil pH was determined in a mixture with a soil/water ratio of 1:5 (wt/vol) using a pH meter (Thermo Orion Star A111; Thermo Fisher, Germany). EC was measured in a mixture with a soil/water ratio of 1:5 (wt/vol) using an EC meter (DDSJ-308A; Leici, China). The soil total carbon (TC) and TN concentrations were measured using a CHNS/O elemental analyzer (FlashSmart; Thermo Fisher, Germany). Ammonia nitrogen (NH_4_^+^-N) and nitrate nitrogen (NO_3_^−^-N) were extracted using 2 M KCl solution. The NH_4_^+^-N concentration was measured using the indophenol blue spectrophotometer method (625 nm). The NO_3_^−^-N concentration was measured using UV spectrophotometer (UV2310II; Tianmei, China). The average and standard deviation (SD) values of soil physicochemical parameters were shown in Table S4 in the supplemental material.

### DNA extraction, 16S rRNA gene amplicon sequencing, and data processing.

Genomic DNA in all samples (0.5 g) was extracted by MP FastDNA spin kit (MP Biomedicals) according to the manufacturer’s instructions. The concentration and integrity of genomic DNA were evaluated using NanoDrop spectrophotometer (ND2000; Thermo Scientific, DE, USA) and agarose gel electrophoresis, respectively. Primers 515F (5′-GTGYCAGCMGCCGCGGTAA-3′) and 806R (5′-GGACTACNVGGGTWTCTAAT-3′) targeting the V4 region of the 16S rRNA gene were used for the amplicon sequencing (48). The PCR system (20 μl) contained 1× FastPfu buffer, 250 μM deoxynucleoside triphosphates (dNTPs), 0.2 μM each primer, 1 U of FastPfu polymerase, and 10 ng of template DNA. PCR was performed in triplicate at 95°C for 3 min, followed by 27 cycles of 95°C for 30 s, 55°C for 30 s, 72°C for 45 s, and a final extension step of 72°C for 10 min. PCR products were purified, pooled with the equimolar concentrations, and sequenced on the Illumina MiSeq PE300 platform at the Majorbio Bio-Pharm Technology Co., Ltd. (Shanghai, China).

Paired-end sequences were merged to a single sequence with length of ∼300 bp using FLASH 1.2.11 (49). The obtained sequences were quality filtered with a maximum expected error of 0.2 and then clustered into OTUs with 97% sequence similarity using USEARCH 10 (50). Representative sequences were classified using RDP Classifier 2.11 (51) and annotated against the SILVA reference database (release 132) with a confidence threshold of 0.8 in QIIME 1.91 (52). Singleton OTUs and OTUs being annotated as plant mitochondria or chloroplast were discarded.

### Metagenomic sequencing and data analysis.

For metagenomic sequencing, libraries were prepared without any amplification step for each sample. Metagenomic shotgun sequencing was performed on the Illumina HiSeq X-10 platform, with 2× 150-bp paired-end reads at the Majorbio Bio-Pharm Technology Co., Ltd. (Shanghai, China). The reads that contain adapters, low quality bases, and 10% of undefined bases were removed. After filtering, clean data were obtained from each sample with a size from 12.1 to 17.7 Gb. High-quality reads were assembled using Megahit version 1.1.2 (https://github.com/voutcn/megahit) with various k-mer sizes. Contigs shorter than 300 bp were discarded. Gene prediction was performed using MetaGene with default parameters. Genes were clustered to remove redundant sequences using CD-Hit (53) at 90% identity and 90% coverage. For taxonomic and functional analysis, the genes were compared (BLASTp) against NCBI-nr, KEGG, and COG databases using DIAMOND (54) with an e-value cutoff of 10^−5^, and only the best hits were retained.

Gene abundances were obtained by mapping the reads of each sample back to the predicted gene sequences using SOAPaligner (55) and calculated transcripts per million (TPM). KEGG ortholog (KO) groups used as functional markers in this study are listed in Table S7 in the supplemental material and include nitrogen and sulfur cycling genes and potential genes involved in plant growth promotion (i.e., genes encoding phosphatase, ACC deaminase, IAA biosynthesis, antioxidant enzymes, and Na^+^/H^+^ antiporter). The relative abundance of genes involved in the nitrogen and sulfur cycles were determined by DiTing (https://github.com/xuechunxu/DiTing) (56).

### Genome binning, taxonomic, and functional annotation.

Reads from triplicate samples were assembled together. MetaWRAP was used to perform assembly, binning, refinement, and reassembly. The qualities (i.e., contamination and completeness) of MAGs were evaluated by CheckM version 1.1.3 (57), and MAGs with a completeness above 50% and contamination lower than 10% were considered for further analysis. The taxonomic classifications were derived using genome taxonomy database GTDB-Tk version 1.3.0 (58). Gene prediction of the MAGs was carried out using the RAST server, and the predicted genes were searched against the SEED subsystems (59) and KEGG database. The genome fold coverage of each MAG was calculated by mapping each of them to the raw reads.

### Statistical analyses.

ANOSIM was used to evaluate the differences among the microbial communities of different rhizocompartments and plant species in PRIMER 6 (60). PERMANOVA was calculated based on Bray-Curtis distance with 999 permutations using the vegan package (61) of R version 3.5.3. PCoA was performed to ordinate the microbial composition in the different samples based on Bray-Curtis distance with the vegan and ggplot2 packages in the R software. OTU counts from bulk soil samples were used as a control to calculate the enrichment (*P *< 0.05; fold change > 2) or depletion (*P *< 0.05; fold change < 0.5) of rhizosphere, rhizoplane, and endosphere, which were illustrated with a volcano plot using TBtools (62). The LEfSe with default parameters (except for LDA value, which was above 3.0) was applied to identify the enriched bacteria of different compartments and plants (63). RDA was performed to determine the relationships between soil properties and microbial community, and the relationships between soil properties and functional category using the vegan package in the R environment.

In this study, the top 20 abundant bacterial genera were used for cooccurrence network construction. The Spearman correlation matrix was calculated (absolute correlation coefficient values > 0.6; *P *< 0.05) using the psych package in the R environment (64). The network was analyzed and visualized using Gephi (version 0.9.2) (65). The mean values of all parameters were derived from at least three biological replicates, and the standard error of the means was calculated. All statistical analyses were performed using *t* tests in GraphPad Prism 7.

### Root exudate collection.

Seeds were surface sterilized with 0.15% mercuric chloride for 10 min and washed 6 times with sterile water. Thereafter, the seeds were germinated in petri dishes with sterile water in a growth chamber at 25°C and 70% relative humidity. Two-day-old seedlings were transferred to self-designed 96-well plates with half-strength Hoagland solution and incubated at 25°C on a 16 h/8 h light/dark cycle. After 7 days, these seedlings were transferred to a 50-ml flask for further growth. The container was covered with aluminum foil to create dark conditions for the roots. The nutrient solution was refreshed every 2 days to minimize potential microbial growth. Root exudates were collected from 4-week-old plants. To collect root exudates, roots were washed using sterile water to remove the attached nutrient solution, and then transferred to a new flask containing 50 ml of sterile water for 1 day. The seedlings were retrieved from the flasks, and the water containing exudates was filtered with 0.22-μm filters to remove root debris and microorganisms. Exudate samples were lyophilized and stored at −80°C.

### Bacterial isolation and growth of *E. meliloti* CL09 in the presence of root exudate.

A total of 50 single rhizobial colonies were isolated from the rhizosphere soil and root of *S. cannabina* using YMA medium (per liter of water, the following were included: 10 g mannitol, 1 g yeast extract, 0.5 g K_2_HPO_4_, 0.2 g MgSO_4_, 0.1 g NaCl, 20 g agar, pH 7.0). Strain CL09 was used to test the growth in the presence of root exudate. Root exudate was dissolved with sterile water to a concentration of 20 mg/ml and filtered with a 0.22-μm filter. YMA plates were supplemented with a final concentration of 0.8 mg/ml root exudate or the same volume of sterile water as a control. The strain CL09 was cultured in yeast mannitol broth until the logarithmic phase. Then, 2.5 μl of culture was spotted on YMA plates and cultivated at 28°C. The colony diameter was measured at 24 h and 48 h. The experiments were conducted with three biological replicates.

### Data availability.

Sequence data for 16S rRNA reads and metagenome have been deposited in the NCBI Sequence Read Archive under BioProject number PRJNA723704. The *Ensifer* genomes generated in this study have been deposited at GenBank under the accession numbers JAHFYY000000000 (MAG46), JAHFYZ000000000 (MAG93), and JAHFZA000000000 (MAG95).
