# Supplementary material for: Patterns in the Microbial Community of Salt-Tolerant Plants and the Functional Genes Associated with Salt Stress Alleviation
Source: Microbiol Spectr. 2021 Oct 27;9(2):e00767-21. doi: 10.1128/Spectrum.00767-21 (PMC8549722; doi:10.1128/Spectrum.00767-21)
Supplement: SUPPLEMENTAL FILE 2 — Supplemental material. Download Spectrum.00767-21-s0001.pdf, PDF file, 1.5 MB [file spectrum.00767-21-s0001.pdf]

Supplementary Data for

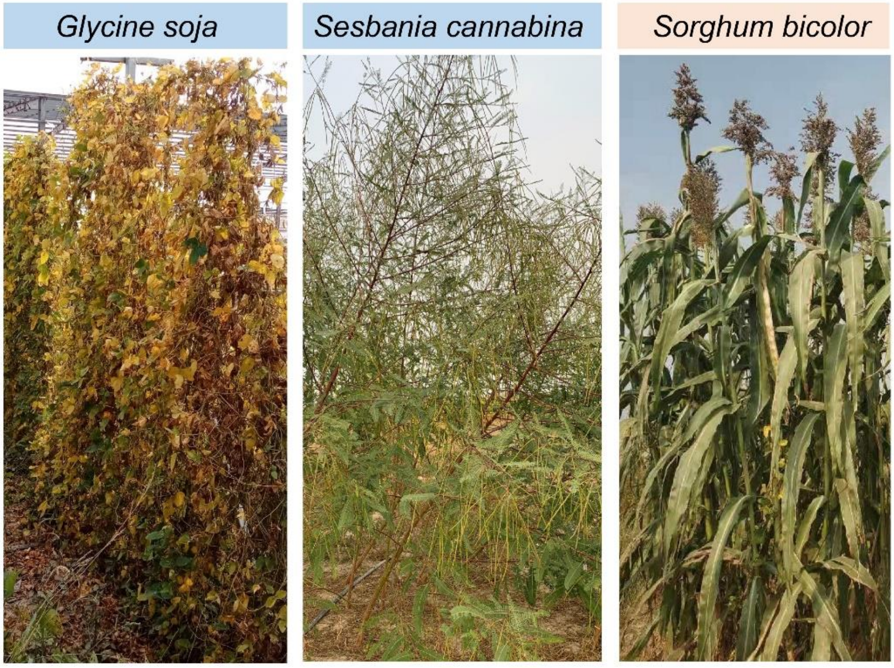

Fig. S1 The growth statuses of three plants when samples were collected

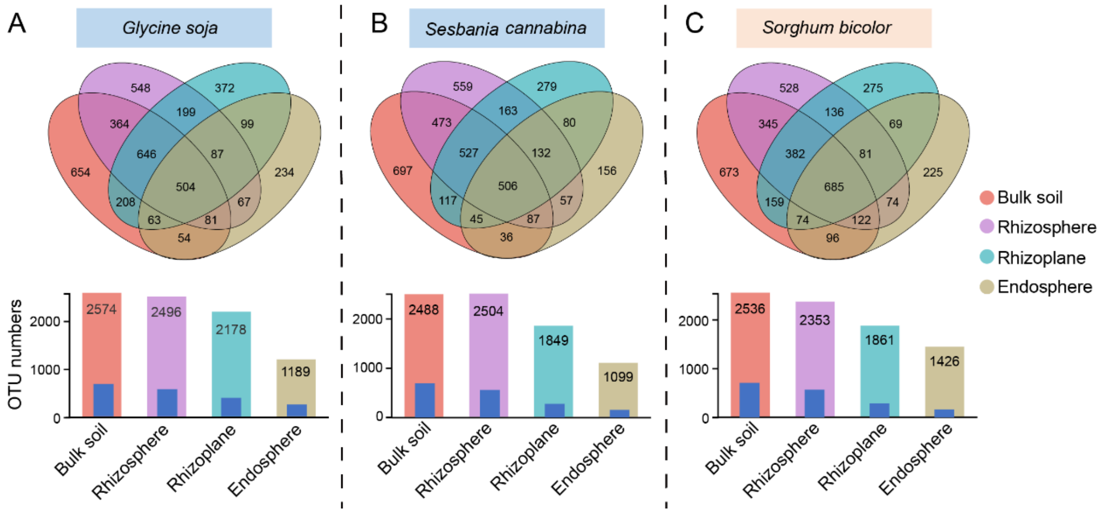

Fig. S2 Venn diagram showing numbers of unique OTU in different compartments

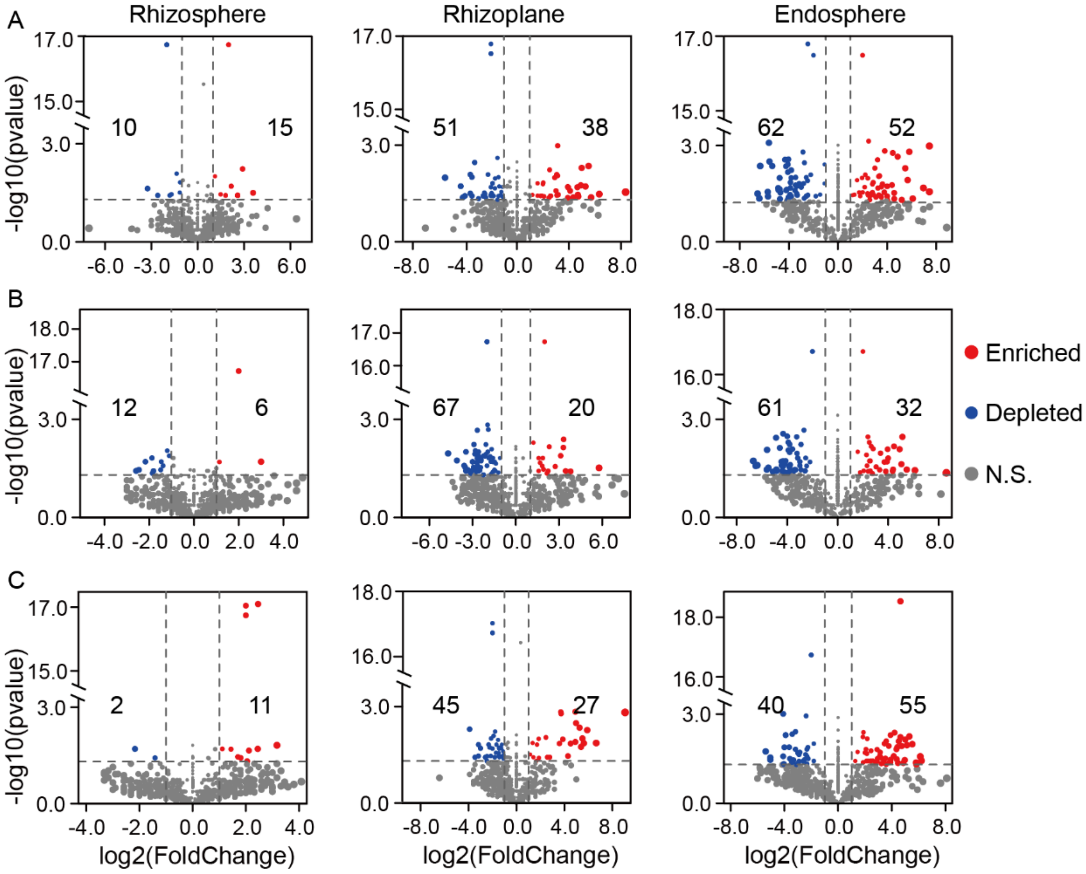

Fig. S3 Enriched and depleted bacterial OTUs in the rhizosphere, rhizoplane and endosphere compartments of *G. soja* (A), *S. cannabina* (B) and *S. bicolor* (C), compared with the bulk soil. Each point indicates an individual OTU, and the red, blue and grey points represent enriched, depleted and no significant different OTUs respectively.

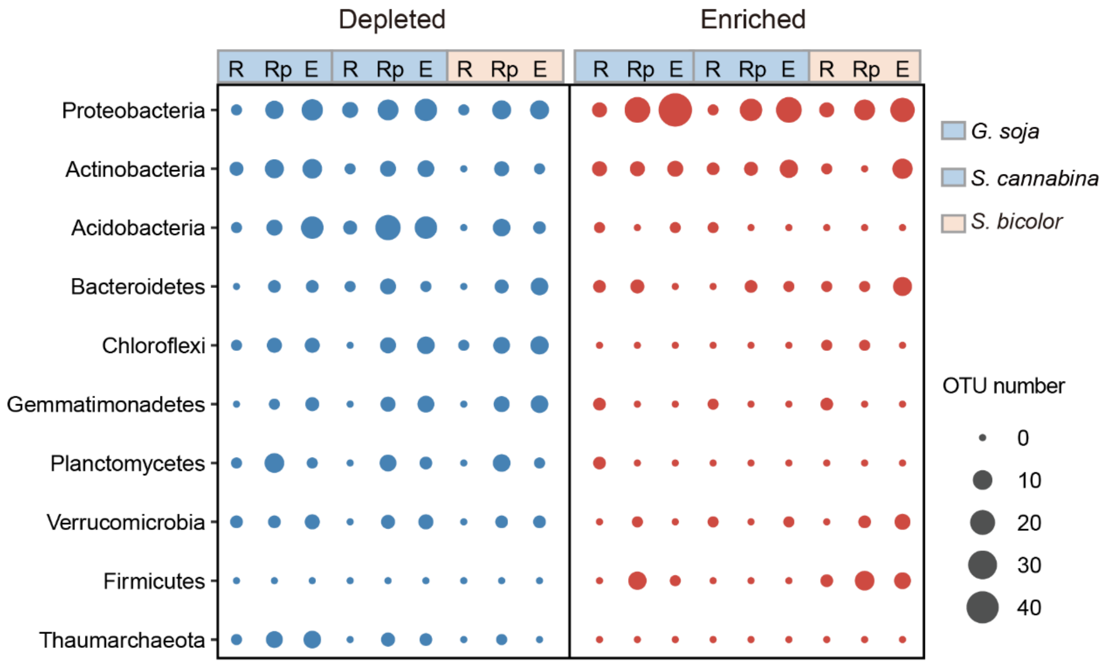

Fig. S4 OTU numbers depleted or enriched at phylum level in rhizosphere (R), rhizoplane (Rp) and endosphere (E) compared to bulk soil.

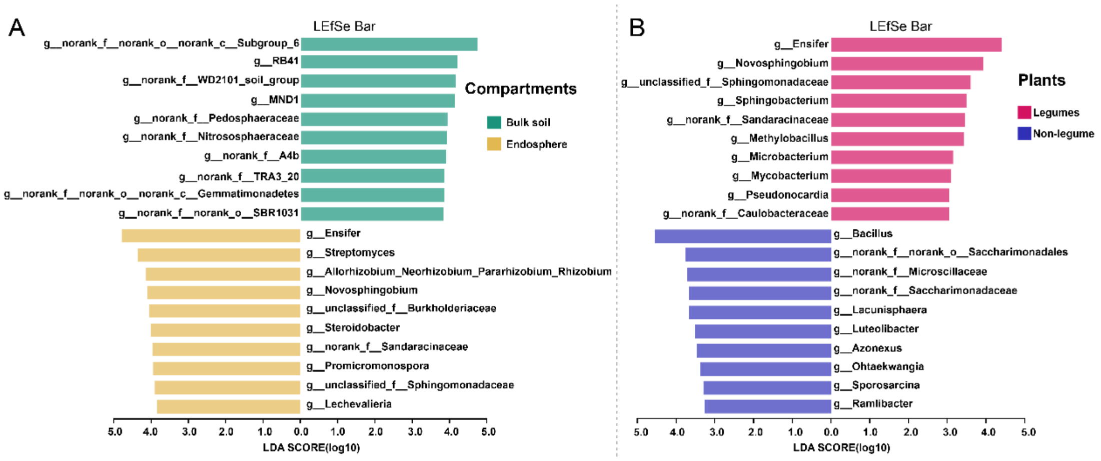

Fig. S5 LEfSe analysis showing the enriched microbes in different compartments (A) and plants (B). Only top 10 most specific biomarker genera were displayed.

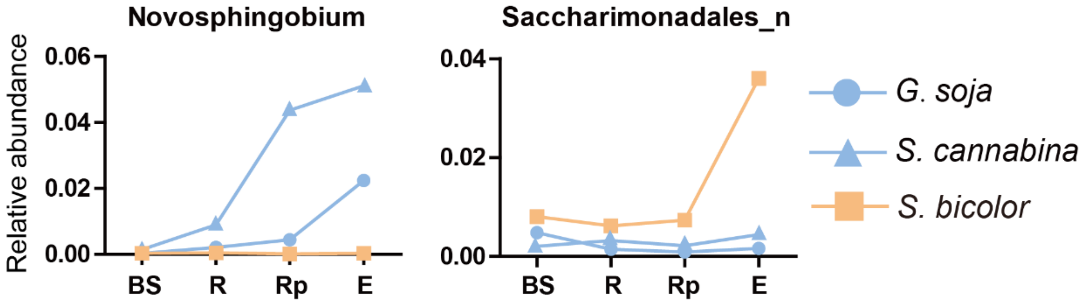

Fig. S6 Relative abundance of *Novosphingobium* and the order of Saccharimonadales among different compartments and plants.

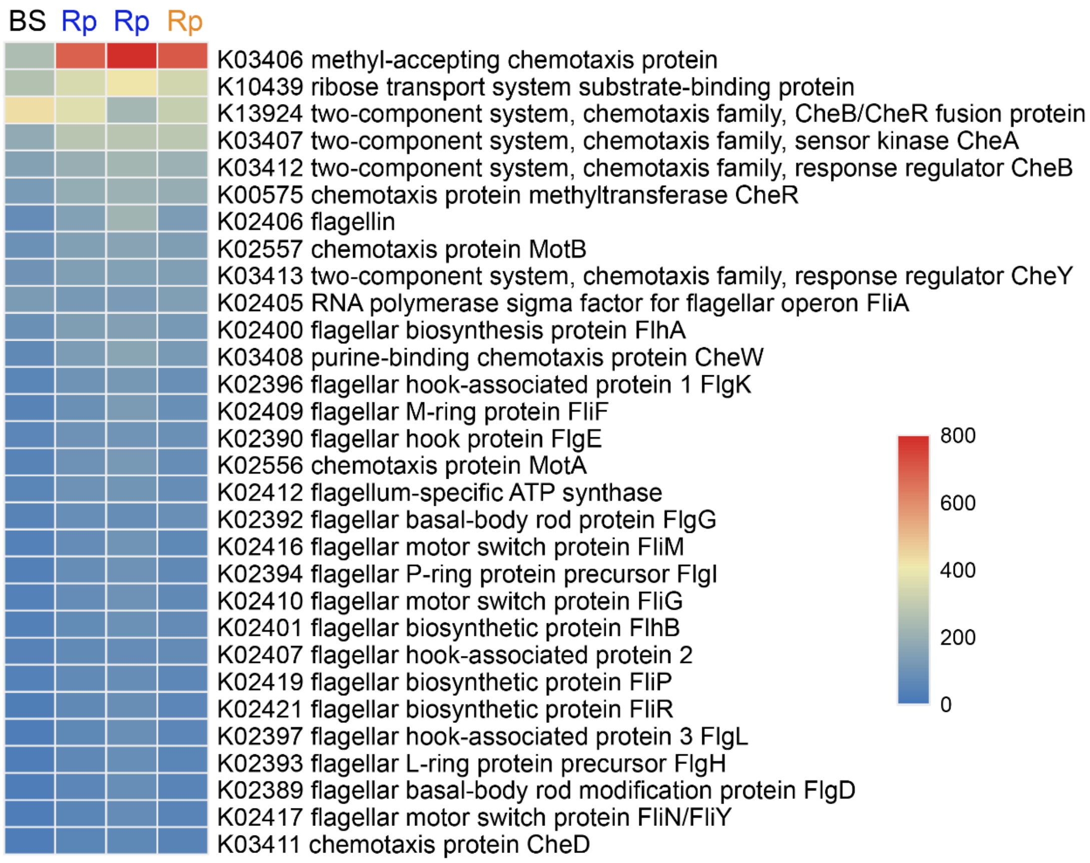

Fig. S7 Heatmap showing the KEGG orthologue group (KO) affiliated with cell motility enriched in the rhizoplane soils. Top 30 abundant KOs are shown. Except for K13924 (enriched in bulk soil) and K02405 (no significant difference), all KOs are significantly represented in rhizoplane soils of three plants ($P < 0.05$). Rhizoplane soils are from *G. soja*, *S. cannabina* and *S. bicolor* in sequence. BS, bulk soil; Rp, rhizoplane.

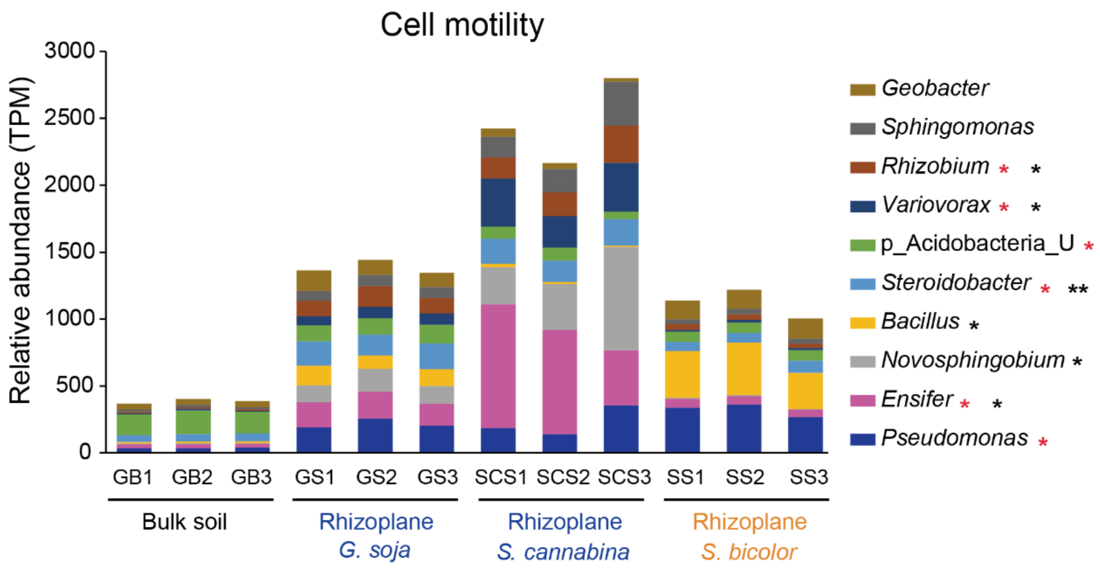

Fig. S8 The source organisms of genes assigned to cell motility. Words in blue mean legume plants. Words in orange mean non-legume plant. Red stars indicate there are significant differences between bulk soil and each rhizoplane soil. Black stars indicate there are significant differences between legume plants and non-legume plant.

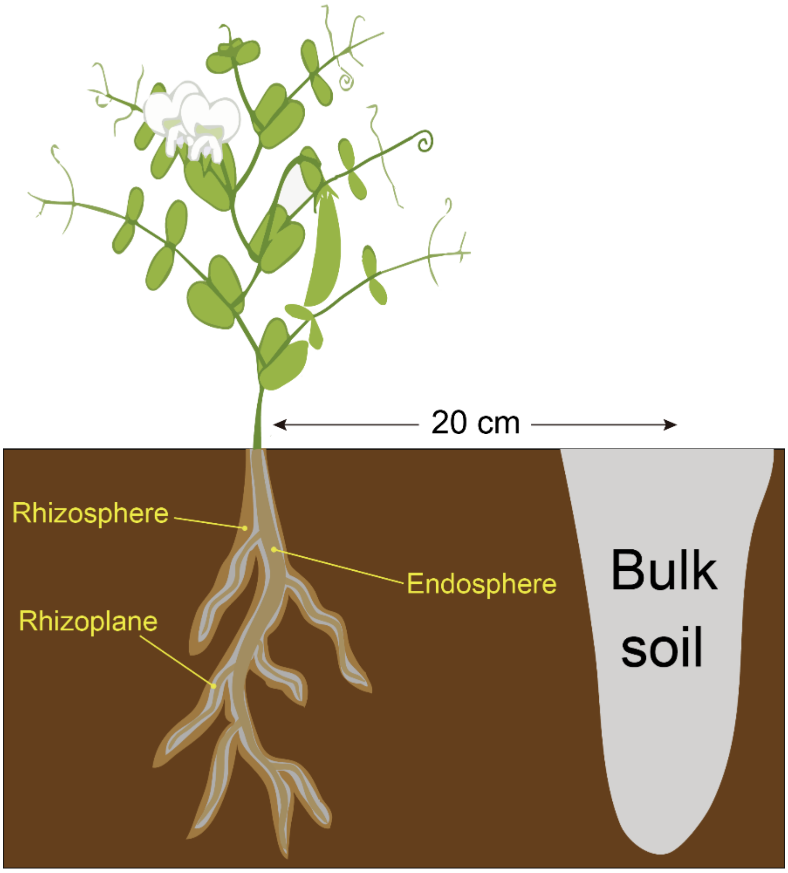

Fig. S9 The schematic diagram showing the locations of the microbial communities sampled

Table S1 The microbial diversity index of all samples. Different letters indicate significantly different groups ($P < 0.05$, Student's t-test).

Plants	Samples	Shannon	Simpson	Ace	Chao 1
G. soja	Bulk soil	6.49±0.18a	0.004±0.001a	3807.02±93.22a	2713.56±148.54a
	Rhizosphere	6.48±0.17a	0.004±0.002a	3520.19±53.26b	2662.45±115.65a
	Rhizoplane	6.09±0.12a	0.009±0.002b	3257.14±59.81c	2270.34±72.75b
	Endosphere	5.01±0.82b	0.009±0.001b	1624.90±247.61d	1219.19±127.97c
S. cannabina	Bulk soil	6.53±0.04a	0.003±0.001a	3718.93±161.18a	2671.89±76.39a
	Rhizosphere	6.59±0.06a	0.003±0.001a	3468.36±296.95a	2675.55±247.49a
	Rhizoplane	5.64±0.37b	0.018±0.004b	2421.69±353.11b	1796.93±344.66b
	Endosphere	4.67±0.75c	0.022±0.002b	1524.92±216.60c	1116.11±127.28c
S. bicolor	Bulk soil	6.52±0.13a	0.003±0.001a	3542.05±371.09a	2604.98±110.65a
	Rhizosphere	6.49±0.04a	0.003±0.000a	3485.21±145.95a	2533.12±114.09a
	Rhizoplane	5.61±0.14b	0.019±0.003b	2984.50±163.45b	2110.82±71.70b
	Endosphere	5.44±0.05b	0.013±0.001c	2151.99±238.46c	1573.31±147.36c

Table S2 PERMANOVA analysis of the microbial community composition of compartment and plant species based on Bray-Curtis distance metric at the OTU level.

Data used	Characteristics	SumsOfSqs	MeanSqs	F_Model	R2 (%)	P_value
Whole data	Compartment	4.01	1.34	11.7	52.31	0.001
	Plant species	0.92	0.46	2.26	12.03	0.021
Bulk soil	Plant species	0.21	0.1	1.09	26.64	0.269
Rhizosphere	Plant species	0.32	0.16	1.48	32.99	0.051
Rhizoplane	Plant species	0.7	0.35	4.09	57.69	0.002
Endosphere	Plant species	1.69	0.84	38.61	92.79	0.004

Table S3 Bacterial abundance (%) in different compartments of *G. soja*, *S. cannabina* and *S. bicolor*. BS, bulk soil; R, rhizosphere; Rp, Rhizoplane; E, Endosphere.

		G. soja				***S. cannabina***				***S. bicolor***			
**Phylum**	**Class**	**BS**	**R**	**Rp**	**E**	**BS**	**R**	**Rp**	**E**	**BS**	**R**	**Rp**	**E**
Proteobacteria	Gammaproteobacteria	10.36	15.36	18.68	25	15.74	14.61	24.67	20.33	13.69	20.48	19.32	21.8
Proteobacteria	Alphaproteobacteria	5.85	7.69	15.43	38.51	7.58	9.14	27.18	40.82	6.78	8.11	6.1	11.23
Actinobacteria	Actinobacteria	12.18	14.03	11.68	12.62	11.76	13.14	16.81	24.97	9.87	9.79	8.87	22.25
Acidobacteria	Subgroup_6	13.27	10.47	8.31	1.13	10.92	11.2	2.64	0.62	11.31	7.14	5.51	1.1
Bacteroidetes	Bacteroidia	4.04	8.29	4.21	3.96	7.15	6.34	6.9	2.66	6.34	7.51	5.48	9.09
Firmicutes	Bacilli	0.45	0.42	10.31	2.55	0.43	0.38	1.58	0.31	0.9	2.57	25.51	10.38
Proteobacteria	Deltaproteobacteria	3.41	4.06	3.66	7.01	3.84	3.98	3.01	3.69	3.6	5.61	2.74	4.16
Gemmatimonadetes	Gemmatimonadetes	4.06	4.67	3.85	0.32	5.86	5.58	3.49	0.33	4.87	7.29	3.47	0.56
Verrucomicrobia	Verrucomicrobiae	3.81	3.38	2.38	1.04	3.2	4.08	1.43	1.09	4.26	3.96	4.63	6.28
Chloroflexi	Anaerolineae	5.39	3.95	2.77	0.42	5.4	3.89	2.33	0.35	5.57	4.6	1.75	0.68
Thaumarchaeota	Nitrososphaeria	6.22	3.95	2.24	0.29	4.16	4.53	0.75	0.29	4.6	3.28	2.63	0.39
Planctomycetes	Phycisphaerae	4.73	3.99	2.72	0.07	3.87	4.11	1.17	0.13	4.49	2.83	1.71	0.27
Acidobacteria	Blastocatellia_Subgroup_4	3.98	3.35	2.27	0.57	4.28	3.91	1.04	0.36	4.69	1.93	1.92	0.42
Planctomycetes	Planctomycetacia	3.75	2.44	1.75	1.46	2.26	2.07	0.48	0.45	3.22	1.38	1.27	0.61
Chloroflexi	Chloroflexia	1.84	1.26	0.97	1.1	1.48	1.31	1.27	0.61	1.72	1.41	0.92	0.91

Table S4 Soil physiochemical parameters of bulk soil and rhizoplane soil. EC, electrical conductivity; TN, total nitrogen; TC, total carbon. Student's *t*-test was performed between bulk soil and rhizoplane soil for each plant, and asterisk was labeled on large value. *$P < 0.05$, ** $P < 0.01$ and *** $P < 0.001$.

Plants	Soil	pH	EC (μs/cm)	NH_4^+-N (mg/kg)	NO_3^--N (mg/kg)	TN (%)	TC (%)
G. soja	Bulk soil	8.77±0.01***	573.33±8.06**	13.52±0.99	6.28±0.67	0.140±0.002	1.91±0.26
	Rhizoplane soil	8.64±0.01	458.00±4.32	21.04±3.54*	8.81±0.63	0.148±0.004*	2.01±0.025*
S. cannabina	Bulk soil	8.86±0.01***	568.00±4.32	6.28±0.78	7.18±3.35*	0.136±0.01	2.01±0.095
	Rhizoplane soil	8.75±0.01	985.33±38.44**	11.17±9.44	6.86±2.32	0.163±0.14*	2.11±0.10
S. bicolor	Bulk soil	8.72±0.03**	612.67±6.18	9.31±0.83	5.63±0.36__	0.136±0.003	1.97±0.06
	Rhizoplane soil	8.52±0.05	956.67±13.89**	11.37±1.39	3.77±0.26	0.142±0.003**	1.95±0.019

Table S5 Details of all the high-quality metagenome assembled genomes (MAGs) constructed from bulk soil and rhizoplane samples. Contigs from triplicate samples were co-assembled. The potential abilities for promoting plant growth were estimated by searching KOs involved in potential pathways for plant growth promotion (listed in Table S7) in all MAGs. “+” indicates this MAG harboured potential plant growth promotion gene. (Table shown in separate file)

Table S6 Subsystem categories of the genome sequence of MAG46, MAG93 and MAG95. MAG46, *E. fredii*; MAG93, *E. alkalisoli*; MAG95, *E. meliloti*.

**Subsystem category**	**MAG46**	**MAG93**	**MAG95**
Cofactors, vitamins, prosthetic groups, pigments	172	85	138
Cell wall and capsule	26	12	34
Virulence, disease and defense	40	58	61
Potassium metabolism	9	9	11
Photosynthesis	0	0	0
Miscellaneous	24	31	38
Phages, prophages, transposable elements, plasmids	0	0	5
Membrane transport	82	111	154
Iron acquisition and metabolism	25	6	22
RNA metabolism	39	39	45
Nucleosides and nucleotides	93	109	116
Protein metabolism	94	130	106
Cell division and cell cycle	0	0	0
Motility and chemotaxis	11	48	63
Regulation and cell signaling	39	32	46
Secondary metabolism	5	0	7
DNA metabolism	94	93	94
Fatty acids, lipids, and isoprenoids	66	53	76
Nitrogen metabolism	37	46	60
Dormancy and sporulation	1	1	2
Respiration	116	161	170
Stress response	87	83	84
Metabolism of aromatic compounds	59	50	42
Amino acids and derivatives	382	296	424
Sulfur metabolism	0	5	13
Phosphorus metabolism	18	21	31
Carbohydrates	251	297	327
Total	1770	1776	2169

Table S7 Full list of KEGG Orthologs (KO) involved in potential pathways for plant growth promotion under saline soil that were searched for in the bulk soil and rhizoplanes metagenome.

Potential pathways for plant growth promotion	KO number	Full gene name
1-Aminocyclopropane-1-carboxylic acid (ACC) deaminase	K01505	acdS; 1-aminocyclopropane-1-carboxylate deaminase
Indole-3-acetic acid (IAA) synthesis	K00466	iaaM; tryptophan 2-monooxygenase
	K04103	ipdC; indolepyruvate decarboxylase
Phosphatase	K00906	aceK; isocitrate dehydrogenase kinase/phosphatase
	K01077	phoA, phoB; alkaline phosphatase
	K01113	phoD; alkaline phosphatase D
	K01078	PHO; acid phosphatase
Siderophore synthesis	K01252	entB, dhbB, vibB, mxcF; bifunctional isochorismate lyase/aryl carrier protein
	K02362	entD; enterobactin synthetase component D
	K02363	entE, dhbE, vibE, mxcE; 2,3-dihydroxybenzoate-AMP ligase
	K02364	entF; enterobactin synthetase component F
	K02552	menF; menaquinone-specific isochorismate synthase
	K04782	pchB; isochorismate pyruvate lyase
	K04783	irp5, ybtE; yersiniabactin salicyl-AMP ligase
	K12239	pchE; dihydroaeruginoic acid synthetase
Antioxidant enzymes	K04564	superoxide dismutase, Fe-Mn family
	K04565	superoxide dismutase, Cu-Zn family
	K00430	peroxidase
	K03781	katE, CAT, catB, srpA; catalase
	K03782	katG; catalase-peroxidase
	K07217	Mn-containing catalase
Exopolysaccharide production	K16566	exoY; exopolysaccharide production protein ExoY

	K16567	exoQ; exopolysaccharide production protein ExoQ
	K16568	exoZ; exopolysaccharide production protein ExoZ
Na^+-transport	K00346	nqrA; Na^+-transporting NADH:ubiquinone oxidoreductase subunit A
	K00347	nqrB; Na^+-transporting NADH:ubiquinone oxidoreductase subunit B
	K00348	nqrC; Na^+-transporting NADH:ubiquinone oxidoreductase subunit C
	K00349	nqrD; Na^+-transporting NADH:ubiquinone oxidoreductase subunit D
	K00350	nqrE; Na^+-transporting NADH:ubiquinone oxidoreductase subunit E
	K00351	nqrF; Na^+-transporting NADH:ubiquinone oxidoreductase subunit F
K^+:H^+ antiporter	K05559	phaA; multicomponent K^+:H^+ antiporter subunit A
	K05560	phaC; multicomponent K^+:H^+ antiporter subunit C
	K05561	phaD; multicomponent K^+:H^+ antiporter subunit D
	K05562	phaE; multicomponent K^+:H^+ antiporter subunit E
	K05563	phaF; multicomponent K^+:H^+ antiporter subunit F
	K05564	phaG; multicomponent K^+:H^+ antiporter subunit G
Na^+:H^+ antiporter	K03313	nhaA; Na^+:H^+ antiporter, NhaA family
	K03314	nhaB; Na^+:H^+ antiporter, NhaB family
	K03315	nhaC; Na^+:H^+ antiporter, NhaC family
	K05565	mnhA, mrpA; multicomponent Na^+:H^+ antiporter subunit A
	K05566	mnhB, mrpB; multicomponent Na^+:H^+ antiporter subunit B
	K05567	mnhC, mrpC; multicomponent Na^+:H^+ antiporter subunit C
	K05568	mnhD, mrpD; multicomponent Na^+:H^+ antiporter subunit D
	K05569	mnhE, mrpE; multicomponent Na^+:H^+ antiporter subunit E
	K05570	mnhF, mrpF; multicomponent Na^+:H^+ antiporter subunit F
	K05571	mnhG, mrpG; multicomponent Na^+:H^+ antiporter subunit G
